# Somatic Mosaicism and Autism Spectrum Disorder

**DOI:** 10.3390/genes12111699

**Published:** 2021-10-26

**Authors:** Alissa M. D’Gama

**Affiliations:** 1 Division of Newborn Medicine, Department of Pediatrics, Boston Children’s Hospital, Boston, MA 02115, USA; alissa.dgama@childrens.harvard.edu; 2 Department of Pediatrics, Harvard Medical School, Boston, MA 02215, USA

**Keywords:** autism spectrum disorder, somatic mosaicism, postzygotic mutation, mosaic variant, next-generation sequencing, genetic diagnosis

## Abstract

Autism spectrum disorder (ASD) is a genetically heterogenous neurodevelopmental disorder. In the early years of next-generation sequencing, de novo germline variants were shown to contribute to ASD risk. These germline mutations are present in all of the cells of an affected individual and can be detected in any tissue, including clinically accessible DNA sources such as blood or saliva. In recent years, studies have also implicated de novo somatic variants in ASD risk. These somatic mutations arise postzygotically and are present in only a subset of the cells of an affected individual. Depending on the developmental time and progenitor cell in which a somatic mutation occurs, it may be detectable in some tissues and not in others. Somatic mutations detectable at relatively low sequencing coverage in clinically accessible tissues are suggested to contribute to 3–5% of simplex ASD diagnoses, and “brain limited” somatic mutations have been identified in postmortem ASD brain tissue. Somatic mutations likely represent the genetic diagnosis in a proportion of otherwise unexplained individuals with ASD, and brain limited somatic mutations can be used as markers to discover risk genes, cell types, brain regions, and cellular pathways important for ASD pathogenesis and to potentially target for therapeutics.

## 1. Introduction

Autism spectrum disorder (ASD) is a common neurodevelopmental disorder characterized by deficits in social interaction and social communication as well as restricted and repetitive patterns of behaviors, interests, and activities. First described in eleven children in 1943 by Dr. Leo Kanner, ASD is estimated to affect between 1 in 54 and 1 in 161 children, with an approximately 4 to 1 male to female ratio and substantial clinical heterogeneity [1,2,3,4,5]. Family and twin studies have demonstrated that ASD has high heritability, and studies over the past decade have demonstrated that ASD has substantial genetic heterogeneity [6].

Although the genetic architecture of ASD continues to be elucidated, multiple types of genetic variants have been shown to contribute to ASD risk. Early genetic causes identified were in individuals with rare monogenic disorders such as tuberous sclerosis, Rett syndrome, and fragile X syndrome, where a substantial proportion of the individuals also have ASD or autistic symptoms, as well as large chromosomal abnormalities identified via karyotype (estimated to each contribute to approximately 3–5% of cases) [7]. An analysis of large cohorts of individuals with ASD in the era of next-generation sequencing (NGS) subsequently revealed contributions of de novo single-nucleotide variants (SNVs) and de novo copy number variants (CNVs) (estimated to contribute to approximately 20–30% of cases), autosomal recessive and X-linked variants (approximately 3–5% of cases), potentially regulatory and noncoding variants (approximately 3–5% of cases), and common variants [6,8,9,10,11,12,13,14,15,16,17,18,19,20,21,22,23,24,25,26,27,28,29]. Although the contributions of rare versus common genetic variants continues to be elucidated, studies have suggested that the common variation contributes significantly to risk on a population level while de novo variation contributes significantly to risk on an individual level, with both types of variation interacting within a given proband [6]. Initially, these studies focused on identifying inherited and de novo germline variants. Recently, we have come to appreciate the role of de novo somatic mosaic variants in ASD and neurodevelopmental diseases more broadly. In this review, we briefly provide an overview of somatic mosaicism in human development and disease, review the literature on somatic mosaicism in ASD, and discuss the implications of somatic mosaicism for ASD diagnosis and therapeutics.

## 2. Somatic Mosaicism, Human Brain Development, and Neurodevelopmental Disease

**Figure 1:**
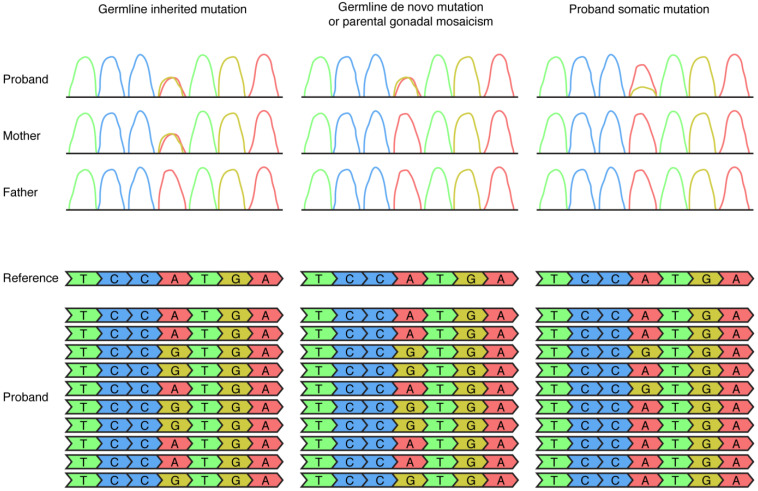
Detection of germline and somatic mosaic mutations by Sanger sequencing (top row) and next-generation sequencing (bottom row) approaches in DNA extracted from clinically accessible tissues such as blood. In this figure the example of an A (depicted in red) to G (depicted in yellow) mutation is used. A germline inherited mutation, in this example a heterozygous mutation inherited from the mother, is detectable as a heterozygous mutation in Sanger sequencing of both the proband and the mother and detectable in 50% of the NGS reads of the proband. A germline de novo mutation or a parental gonadal mosaic mutation is detectable as a heterozygous mutation in Sanger sequencing of the proband, not detectable in Sanger sequencing of the parents, and detectable in 50% of the NGS reads in the proband. A somatic mutation is sometimes detectable in Sanger sequencing of the proband (in this example, as the small yellow peak) and is detectable in <50% of the NGS reads (in this example, 20% of the NGS reads) in the proband.

Traditionally, human genetics studies focused on identifying germline mutations in human diseases. A germline mutation is present in all of the cells of an affected individual and can be either inherited, which means it is detectable in one or both parents of the affected individual, or de novo, which means it is not detectable in either parent of the affected individual. A de novo mutation generally arises during oogenesis or spermatogenesis in the mother or father, respectively, of the affected individual. Since a germline mutation is present in all of the cells of an affected individual, it can be detected in DNA extracted from any tissue of that individual, including clinically accessible tissues such as blood or saliva. As mentioned above, de novo germline mutations in many genes have been shown to significantly contribute to ASD risk (estimated that de novo mutations contribute to ASD risk in approximately 30% of simplex autism), which can make sense, given that many genes are important for brain development and individuals with ASD often do not reproduce, requiring de novo mutations to continually occur to maintain ASD incidence rates [30,31]. In the past decade, human genetics studies have provided mounting evidence for the role of de novo somatic mosaic mutations in noncancerous human diseases, especially neurodevelopmental diseases [32,33,34]. A somatic mosaic mutation arises postzygotically and is present only in the daughter cells of the originally mutated cell. If the somatic mutation arises relatively early in development before gastrulation, it can be detected in cells from all three germ layers (ectoderm such as brain tissue and epithelial cells in saliva, mesoderm such as blood, and endoderm). If the somatic mutation arises relatively late in development after gastrulation or after neurulation, it can be detected only in cells from one germ layer or the nervous system, respectively. Technically, a somatic mosaic mutation is present only in a subset of somatic cells, a gonadal mosaic mutation is present only in a subset of germ cells, and a gonosomal mosaic mutation is present in a subset of somatic and gonadal cells. Practically, this is difficult to determine, and this review uses somatic mutation to refer to a postzygotic mutation present in a subset of the cells of an affected individual (Figure 1).

The human body, including the human brain, accumulates somatic mutations postzygotically during embryonic development and throughout postnatal life. Studies have demonstrated relatively high rates of somatic mutations during embryogenesis and particularly neurogenesis (approximately 5.1 SNVs per progenitor per day), with continued accumulation of somatic mutations approximately linearly in neurons during postnatal life (approximately 23 SNVs per neuron per year in the prefrontal cortex) [35,36]. Somatic mutations that have a damaging effect, occur at an appropriate time during embryonic development, and occur in an appropriate progenitor cell (e.g., neural progenitor cell), have the potential to contribute to neurodevelopmental disease through several mechanisms. In obligatory somatic mutation diseases, the mutation in the germline state is incompatible with life, and thus, the mutation is only detected in the somatic state. For example, hemimegalencephaly, characterized by the abnormal enlargement of a cerebral hemisphere, is caused by obligatory somatic mutations that abnormally activate the mammalian target of rapamycin (mTOR) pathway, such as gain-of-function mutations in *MTOR* or its upstream positive regulator *AKT3* as well as loss-of-function mutations in its upstream negative regulator *DEPDC5* [37,38,39,40]. In the “two hit” model, first described by Dr. Alfred Knudson for retinoblastoma, an affected individual has a germline mutation in one allele of a gene, and a somatic mutation occurs in the second allele of that gene, classically leading to cancer or overgrowth manifestations [41]. For example, in neurofibromatosis type 1, an affected individual has a germline mutation in one NF1 allele, and somatic mutations in the second NF1 allele lead to neurofibromas [42]. In some diseases, both germline and somatic mutations have been identified, and there is often a relationship between the percent of cells affected by a mutation and the severity of the phenotype; i.e., somatic mutations, which by definition affect a smaller percentage of cells than germline mutations, are associated with milder phenotypes. While the mechanisms by which somatic mutations contribute to ASD risk continue to be elucidated, recent studies discussed in detail below suggest multiple processes may contribute, with one or more germline and/or somatic mutations contributing to ASD risk in an affected individual.

## 3. Somatic Mosaicism and ASD

Similarly to the initial evidence for germline mutations contributing to ASD risk, the initial evidence for somatic mutations contributing to ASD risk came from studies that identified somatic mutations in rare monogenic conditions associated with ASD or autistic symptoms. A somatic mutation in *MECP2* on the X chromosome was first identified in 2000 in a male with Rett syndrome, a neurodevelopmental disorder that primarily affects females and is usually lethal in males [43]. Evidence of somatic mosaicism in females with Rett syndrome was demonstrated the following year, with the authors noting that it was difficult to detect low levels of mosaicism with the available DNA sequencing methods [44]. Studies from this time period also demonstrated evidence of parental somatic and gonadal mosaicism in associated disorders, with one study showing parental mosaicism in 6/62 families with *TSC1* or *TSC2* mutations, which has important implications for genetic counseling [45]. An investigation of monozygotic twin brothers with Fragile X Syndrome demonstrated a relationship between the percent of cells affected by the CGG repeat mutation and the severity of the phenotype, with the brother with a full mutation in all of his cells having more severe intellectual disability than the brother with mosaicism for a premutation and a full mutation [46].

Early studies also included case reports of somatic mutations detected in patients with ASD, mainly chromosomal or copy number variation. These included a male with ASD, a coloboma, and a mosaic ring chromosome 14 (2/3 of cells with partial trisomy and 1/3 of cells with partial monosomy of proximal chromosome 14); a child with ASD and mosaic trisomy of chromosome 8; a female with ASD and mosaic duplication of chromosome 4p; a male with ASD, moderate to severe intellectual disability, and myoclonic epilepsy and mosaic deletion of the terminal end of chromosome 20 (8% of lymphocyte cells); a female with ASD, NF1, and a mosaic ring chromosome 17; a male with ASD, intellectual disability, and mosaic tetrasomy of chromosome 3q; and a female with mosaic duplication of chromosome 2p25.3 (33–39% of her lymphoblastoid cells) who passed the duplication to two male half siblings with ASD [47,48,49,50,51,52,53]. In addition, there was one larger study of 116 males with ASD that reported 16% demonstrated mosaic aneuploidy in cultured peripheral blood cells [54]. These chromosomal abnormalities and the level of mosaicism were mainly detected using cytogenetic techniques, including karyotyping and fluorescence in situ hybridization, as well as a chromosomal microarray.

With the advent of NGS, the initial WES studies in large ASD cohorts focused on detecting de novo germline mutations; however, some commented on potential somatic mutations. O’Roak and colleagues performed WES for 209 simplex families from the Simons Simplex Collection (SSC) and reported two families with likely paternal germline mosaicism and nine probands with variant allele frequencies (VAFs) suggestive of somatic mosaicism, accounting for 4.2% of total de novo mutations [21]. A follow-up study targeting 64 candidate risk genes in almost 3500 probands from the SSC and The Autism Simplex Collection noted events in three probands and two siblings with VAFs suggestive of somatic mosaicism and one instance of maternal mosaicism [19]. Iossifov and colleagues performed WES on 343 simplex families from the SSC and reported two examples of likely paternal mosaicism, where deep sequencing of PCR showed the variants present at low VAFs not seen in the relatively low coverages WES data [12]. The authors of these initial WES studies noted that the bioinformatic pipelines used were not optimized for the detection of somatic mosaicism and likely filtered out both proband and parental somatic mutations as false-positive sequencing errors, which are admittedly difficult to discriminate at standard WES coverage.

In the past five years, multiple groups have systematically reanalyzed WES data from large ASD cohorts using improved bioinformatics pipelines and validation methods to detect somatic mutations and estimate the contribution of somatic mutations to ASD risk. These estimates are derived from mathematical models which take into account factors including baseline mutation rates, differences between probands and controls, error rates, and types of mutations. Freed and Pevsner analyzed WES data from 2388 simplex families from the SSC and reported that 5.4% of total de novo mutations appeared to be somatic [55]. All classes of somatic mutations were enriched in probands compared to unaffected siblings. They estimated that 33% of somatic mutations in probands contributed to 5.1% of ASD diagnoses in simplex families. Dou and colleagues analyzed WES data from 2361 simplex families from the SSC and reported that 65.8% of missense and loss-of-function (LOF) somatic mutations with high VAFs ≥ 20% in probands as well as 53.4% of parental missense and LOF somatic mutations with low VAFs < 20% transmitted to probands increased the risk of ASD by approximately 6% total, 3.4%, and 2.6%, respectively [56]. They noted that the somatic mutations detected were enriched in LOF-constrained exons. Lim and colleagues analyzed 5947 families from the SSC as well as the Autism Sequencing Consortium (ASC) and reported that 7.5% of total de novo mutations appeared to be somatic [57]. Damaging nonsynonymous mutations in critical exons of prenatally brain-expressed genes were enriched in probands compared to controls. Krupp and colleagues analyzed 2264 simplex families from the SSC and reported that 22% of total de novo mutations appeared to be somatic and 6.8% of presumed de novo mutations in probands appeared to be somatic in a parent [58]. Synonymous somatic mutations were enriched in probands compared to controls. They suggest some of the impact of synonymous mutations may be via splicing effects, and this is an area that remains to be elucidated in future functional studies. They estimated that somatic mutations contributed risk to 3–4% of simplex ASD, noting approximately 2% of that risk from synonymous mutations.

Overall, these four studies demonstrated that somatic mutations in probands and in parents transmitted to probands contribute to ASD risk in approximately 3–5% of simplex families. This is likely an underestimate of the true contribution of somatic mutations to ASD risk, as the studies analyzed relatively low-coverage WES data and were limited to detecting somatic mutations present in clinically accessible DNA sources (mainly whole blood, as well as saliva and lymphoblastoid cell lines). The somatic mutations detected revealed new ASD risk genes and provided insights into brain regions that may be important for ASD pathogenesis; for example, Dou and colleagues noted that the new ASD risk genes tended to have higher expression in the cerebellar hemispheres, and Lim and colleagues noted that genes with somatic mutations in critical exons expressed during prenatal brain development were enriched for expression in the amygdala [56,57]. All four studies used WES data from the SSC; however, the authors reported different rates and burden analyses of somatic mutations. These differences likely result from varied computational and validation approaches, including differences in target regions, quality thresholds, calls based on reanalysis only versus requiring overlap with original analysis, validation methods, and predictive models. Moving forward, these studies highlight the need for both improved bioinformatics pipelines and deeper sequencing to detect somatic mutations accurately and comprehensively [59].

Recent studies have expanded from focusing on the contribution of somatic small-scale (mainly single nucleotide) variants in the exome to analyzing the contribution of somatic mosaicism from CNVs and all variants in the genome. Yuen and colleagues performed whole-genome sequencing (WGS) on 200 simplex families with an average depth of 32X and reported that a portion of de novo mutations had VAFs < 33% and appeared to be somatic, finding 3.19 somatic mutations per genome and 0.036 per exome [60]. They estimated that 1.1% of de novo mutations presumed to be germline were likely somatic. An analysis of 13 monozygotic twins discordant for ASD did not detect any somatic CNVs in the twin pairs, noting that the analysis was limited to clinically accessible saliva samples [61]. A large study by Sherman and colleagues that analyzed genotype array intensity data from 12,077 probands with ASD from the SSC, and the Simons Powering Autism Research for Knowledge (SPARK) datasets reported a significant burden of large (>4 Mb) somatic CNVs in probands compared to unaffected siblings [62]. They found that 0.4% of probands, and 0.2% of siblings carried a somatic CNV and noted that larger size of somatic CNVs in probands correlated with increased ASD severity. Interestingly, CNVs recurrently observed in the de novo germline state in individuals with ASD, such as 16p11.2, were not detected in the somatic state. Taken together, these studies provide evidence that somatic mutations of various types contribute to ASD risk. Future studies are needed to better understand the contribution of structural and especially noncoding somatic variants. 

The NGS studies discussed above used DNA extracted from clinically accessible tissues, mainly blood or saliva. However, a damaging somatic mutation has the potential to occur at any developmental time and in any progenitor cell and, as has been demonstrated for other neurodevelopmental disorders, can occur late enough during embryonic development to be limited to the brain and not be able to be detected in the blood. Generally, such somatic mutations are not expected to lead to visible lesions in ASD brain, which makes the detection of the mutations and interpretation of their functional consequences more challenging. In some individuals with ASD, “patches” of disorganization have been identified in the prefrontal and temporal cortex, and it has been hypothesized that these patches may represent visible consequences of somatic mutations [63,64]. Several recent studies have used DNA extracted from postmortem brain tissue from individuals with ASD to detect somatic mutations. An initial study of 55 postmortem ASD brains analyzed deep sequencing data across 78 candidate genes and reported deleterious somatic mutations in two individuals with ASD and one individual with a fragile X premutation [65]. The authors showed that some of these somatic mutations were regionally distributed within the brain. A subsequent study by Rodin and colleagues of 59 postmortem ASD brains and 15 control brains analyzed deep WGS data (approximately 250X) for somatic SNVs and reported that somatic mutations in neural enhancer sequences were enriched in ASD brains compared to control brains [66]. The group also reported detection of two somatic CNVs in postmortem ASD brains, including one complex CNV shown to be present in both neuronal and non-neuronal cells [62]. These studies provide growing evidence that somatic mutations, some limited to the brain, contribute to ASD risk. While postmortem ASD brain tissue is a scare and precious resource, the consideration of including brain tissue and of collecting nonbrain tissue alongside brain tissue in future studies of somatic mosaicism in ASD is critical [67]. The main NGS studies investigating somatic mosaicism in ASD are summarized in Table 1.

## 4. Diagnostic and Therapeutic Implications

As discussed above, multiple studies over the past several years have demonstrated that somatic mutations of various types contribute to ASD risk. Detecting somatic mutations should ideally be part of the diagnostic genetic testing for an individual with ASD to paint as full a picture as possible of the genetic variants in an affected individual contributing to ASD risk. In some cases, a somatic mutation may be the main pathogenic variant in an affected individual. In these cases, the standard genetic testing for germline mutations is negative, and identifying that somatic mutation ends an otherwise unexplained diagnostic odyssey. In other cases, a combination of germline and somatic variants may lead to ASD in an affected individual. In addition, the identification of a somatic mutation has important implications for reproductive counseling. If a presumed de novo germline mutation in an individual with ASD is in fact a somatic mutation, the recurrence risk for future children of the parents is similar to the risk in the general population. On the other hand, if a presumed de novo germline mutation in an individual with ASD is in fact due to mosaicism in one of the parents, the recurrence risk for future children of the parents is increased compared to the risk in the general population.

However, several barriers currently exist to detecting somatic mutations in clinical testing. Firstly, even if a contributory somatic mutation occurs early enough to be detectable in clinically accessible tissues such as blood, current clinical genetic testing is generally not technically optimized to detect and validate somatic mutations and thus to report somatic mutations. Since a somatic mutation occurs in only a subset of the cells of an affected individual, a relatively high sequencing depth, ideally >500X, and special bioinformatics pipelines are needed to confidently detect a low-frequency somatic mutation and discriminate it from a false-positive sequencing error. Currently, WES (and in some cases WGS) is becoming integrated into clinical practice, and research consortia such as the SSC and ASC that enroll individuals with ASD are important genetic diagnostic avenues to continue elucidating the landscape of somatic mutations contributing to ASD risk through deep NGS studies while waiting for deeper NGS to become clinically integrated. Secondly, if a contributory somatic mutation occurs late enough to only be detectable in brain tissue, it is currently not possible to detect that mutation on either a clinical or genetic basis in a living individual affected with ASD, as neurosurgery to access brain tissue is generally not performed except in some individuals with refractory epilepsy or brain tumors. Ongoing research in the neuro-oncology and epilepsy fields is investigating the potential of cell free DNA in the CSF as a source of DNA that could be used to detect brain limited somatic mutations [68]. Thirdly, the threshold for disease remains unclear. Studies from the epilepsy field have shown that somatic mutations as low as 1% variant allele frequency (VAF) can cause seizures and abnormal brain development [69]. Future studies of somatic mutations in normal and affected individuals will hopefully identify such thresholds, which may depend on the specific gene and type of mutation.

Understanding somatic mosaicism in ASD also has implications for the development of therapeutics. Since somatic mutations by definition occur in only a subset of cells, identifying somatic mutations associated with ASD that are restricted to or enriched in particular cell types, brain regions, neural circuits, and/or molecular pathways then suggests that those areas may be important in ASD pathogenesis. For example, the studies discussed above that analyzed WES for evidence of somatic mosaicism associated with ASD risk suggested roles for the cerebellar hemispheres and the amygdala. A somatic mutation may lead to milder or different manifestations than the corresponding germline mutation or a mutation may be too damaging to occur in the germline state, and identification in the somatic state may lead to the identification of new ASD risk genes or chromosomal regions. In addition, a somatic mutation may modify the effects of a germline mutation and lead to different manifestations than the germline mutation alone. As more individuals with ASD are sequenced and more risk genes emerge, genotype–phenotype studies have the power to identify ASD subtypes and implicate molecular mechanisms and cellular pathways for potential targeted treatments, and it is critical to identify both germline and somatic mutations to accurately analyze these relationships. Moreover, studying such somatic mutations may help classify subtypes of ASD and provide a potential explanation for individuals with ASD who are high functioning in some areas yet severely deficient in others.

## 5. Conclusions

The genetic architecture of ASD is heterogeneous—a puzzle with many pieces—and growing evidence over the past decade suggests that somatic mutations represent one of the missing pieces of the puzzle. Initially detected in rare monogenic syndromes associated with ASD, somatic mutations have now been shown to also contribute to nonsyndromic ASD risk. Multiple NGS studies using DNA extracted from clinically accessible sources have demonstrated that somatic mutations contribute to 3–5% of simplex ASD risk. This is likely an underestimate of the true contribution of somatic mutations to ASD risk given that (1) these studies used relatively low depth of coverage limiting detection of somatic mutations with low VAFs, and (2) NGS studies using DNA extracted from postmortem ASD brain tissue have detected “brain limited” somatic mutations. Future studies are needed to further investigate somatic mutations that can be detected using deep depth of coverage in clinically accessible DNA sources and to develop innovative methods to detect “brain limited” somatic mutations. Advances in sequencing and bioinformatics have been critical to somatic mosaicism studies over the past decade. Continued collaboration, such as the ASC, SSC, and Brain Somatic Mosaicism Network, will be needed to increase the power to detect somatic mosaicism in ASD and to develop consensus calling pipelines for somatic mutations in human disease [70]. In the next decade, it will be important to integrate somatic mutation detection into clinical genetic testing and use somatic mutations as “markers” to elucidate neural circuits critical to ASD pathogenesis, with the ultimate goal of using the knowledge to develop targeted therapeutics.

## Figures and Tables

**Table 1:** Summary of studies analyzing NGS data for somatic mosaicism in ASD.

Study	Subjects	DNA Source	Genetic Testing	Main Findings
O’Roak et al. [21]	209 SSC families	Blood	WES	Somatic mutations accounted for 4.2% of de novo mutations
Freed et al. [55]	2388 SSC families	Majority blood	WES reanalysis	Somatic mutations accounted for 5.4% of de novo mutations, contribute to 5.1% of simplex ASD risk
Dou et al. [56]	2361 SSC families	Majority blood	WES reanalysis	Missense and LOF somatic mutations with VAF ≥ 20% in probands and with VAF < 20% in parents transmitted to probands contribute to 3.4% and 2.6%, respectively, of simplex ASD risk
Lim et al. [57]	5947 SSC and ASC families	Majority blood	WES reanalysis	Somatic mutations accounted for 7.5% of de novo mutations
Krupp et al. [58]	2264 SSC families	Majority blood	WES reanalysis	Somatic mutations accounted for 22% of de novo mutations, and contribute to 3–4% of simplex ASD risk
Yuen et al. [60]	200 simplex families	Majority blood	WGS	Somatic mutations accounted for 1.1% of de novo mutations
Sherman et al. [62]	12,077 probands and 5500 unaffected siblings 60 probands	Blood or saliva Postmortem brain tissue	Genotype arrays Deep WGS	Probands had a significant burden of large (>4 Mb) somatic CNVs compared to controls Detected somatic CNVs in ASD brain (nonbrain tissue not available)
D’Gama et al. [65]	55 probands and 50 controls	Postmortem brain tissue	Deep targeted NGS	Detected deleterious somatic SNVs in ASD brain
Rodin et al. [66]	59 probands and 15 controls	Postmortem brain tissue	Deep WGS	Detected landscape of somatic SNVs in ASD brain
